# Alumni

**Published:** 1879-02

**Authors:** 


### ALUMNI.

Arrangements for the meeting of the alumni of the Ohio Dental College, already announced elsewhere, have been about completed. The organization will be effected on Thursday, March 6th, at two o'clock p. m., when the historical address will be delivered by Dr. Geo. Watt. In the evening at eight o'clock class reports will be given, and such other matters presented, as shall be appropriate to the occasion.

It is desirable that every regular graduate of the institution be present, so far as possible.

